# Short courses on maternal mental health provided by healthcare providers in low- and middle-income countries: a scoping review

**DOI:** 10.3389/fmed.2026.1950277

**Published:** 2026-10-08

**Authors:** Geldine Chironda, Catherine Titanus Mselema, Maryam Rezaeianjam, Angela R. Lopatka, Chank Mwalweni, Wendy Huynh, Sylvester Shallon Malunga, Esmie Mkwinda, Clariana Ramos de Oliveira

**Affiliations:** 1 Health Sciences, Saint John of God University, Mzuzu, Malawi; 2 School of Public Health, University of Nevada Las Vegas, Las Vegas, NV, United States; 3 School of Nursing, University of Nevada Las Vegas, Las Vegas, NV, United States

**Keywords:** health professions education, healthcare providers, low- and middle-income countries, maternal mental health, perinatal mental health, scoping review, training

## Abstract

**Introduction:**

Maternal mental health (MMH) conditions are increasingly alarming yet remains under recognized and poorly integrated into healthcare services, particularly in low- and middle-income countries (LMICs). Nurses, midwives, and other frontline healthcare providers are well positioned to identify, support, and refer women with perinatal mental health conditions. However, the content and delivery of short MMH training courses for healthcare providers in LMICs are not well documented. This scoping review mapped the available evidence on short MMH training courses for healthcare providers in LMICs.

**Methods:**

This scoping review followed the Joanna Briggs Institute (JBI) methodology and was registered with the Open Science Framework. Reporting adhered to the PRISMA Extension for Scoping Reviews (PRISMA-ScR). A comprehensive literature search was conducted from PubMed, CINAHL EBSCO Host, Web of Science, Scopus, African Journals online (AJOL) and Sabinet with EndNote and Rayyan used to manage records, screening, and study selection.

**Results:**

Fifteen studies met the inclusion criteria. Most training programmes targeted nurses, midwives, primary healthcare workers, or community health workers and were implemented after 2020. Course content focused primarily on two areas: (1) clinical knowledge and screening for perinatal mental disorders using validated tools such as the Edinburgh Postnatal Depression Scale (EPDS), Patient Health Questionnaire-9 (PHQ-9), and Generalized Anxiety Disorder-7 (GAD-7); and (2) counselling and psychosocial support, including psychoeducation, empathic communication, problem-solving, and family-centred care. Evaluation methods predominantly assessed knowledge, competence, and self-efficacy through pre- and post-training assessments. Nine studies reported significant improvements in participants’ knowledge and skills, four demonstrated improved screening and referral practices, three reported positive clinical outcomes for women, and three showed successful integration of MMH training into routine health services.

**Conclusion:**

Short MMH training courses in LMICs primarily strengthen healthcare providers’ knowledge, screening, and counselling competencies. Although evidence suggests improvements in provider capacity and service delivery, more research is needed to evaluate long-term behavioural, clinical, and health system outcomes.

## 1 Introduction

Maternal mental health (MMH) is a fundamental component of maternal and child health and a major global public health priority (1, 2), yet it remains under recognized and under-integrated into healthcare practices, particularly in low- and middle-income countries (LMICs). Worldwide about 10% of pregnant women and 13% of women who have just given birth experience a mental disorder, primarily depression (3). Untreated maternal mental health conditions are associated with adverse maternal outcomes, including increased risk of suicide, poor quality of life, impaired functioning, and reduced engagement with health services (4). They also have important consequences for children, contributing to suboptimal infant caregiving, impaired mother–infant bonding, and poorer cognitive, emotional, and developmental outcomes (5–8).

Because specialist mental health services remain scarce in many LMICs, integrating maternal mental healthcare into routine maternal, newborn, and primary healthcare has become a global priority (9). Nurses, midwives, physicians, community health workers, and other frontline healthcare providers are often the first, and sometimes only,professionals with whom women interact during pregnancy and the postpartum period period (9–11). These providers are well positioned to identify psychological distress, provide evidence-informed psychosocial support, deliver brief interventions, and facilitate timely referral when appropriate. However, their capacity to perform these roles depends on access to adequate education and continuing professional training.

Despite growing recognition of the importance of maternal mental health, healthcare providers in many LMICs continue to report limited knowledge, low confidence, and insufficient preparation to recognize and manage common perinatal mental health conditions (12, 13). The WHO's 2016 (14) global strategy for women's, children's, and adolescents’ health emphasized the importance of strengthening health systems to better support mental health during the maternal period, including through improved training of healthcare providers (14). However, the extent to which healthcare professions equips maternal mental health short courses for additional knowledge remains unclear.

Perinatal mental health disorders such as depression, psychosis and anxiety, affect a significant number of women globally, with approximately 20% in low- and middle-income countries (LMICs) experiencing these conditions during the postpartum period (15–17). Mothers experiencing these challenges are at heightened risk for suicide, difficulties in maternal-infant bonding, and inadequate caregiving, which can adversely affect the cognitive, socio-emotional, and psychomotor development of infants (6–8, 18). Despite these challenges there is a gap in screening, diagnosis and reporting of maternal mental health conditions in LMICs (19).

In addition, lack of training on maternal mental health modules in healthcare programs resulting to limited availability of trained mental health professionals further exacerbating the issue (2). Perinatal mental health is an important aspect that has frequently been neglected in maternal and newborn care programs. Incorporating maternal mental health courses in health programs can help develop a health workforce skilled in the timely identification, screening, and management of maternal mental health problems (20, 21).

Nevertheless, it is necessary to gain a clear understanding of how maternal mental health topics are currently included in training for healthcare providers. This scoping review mapped the evidence on the nature of maternal mental health courses for health professions education across low and middle income regions. By identifying the gaps in education and training, this review will provide insights that can inform integration of maternal mental health into curriculum development, advocate for policy changes, and enhance maternal mental health outcomes globally.

### 1.1 Main review question

What evidence exist on maternal mental health short courses provided by healthcare providers in low- and middle-income countries?

### 1.2 Specific review questions

1. What are the publication characteristics of studies?
2. Which health professionals are teaching maternal mental health courses?
3. What are the characteristics of mental health care courses being taught be healthcare professionals?
4. How are the courses evaluated?
5. What are the outcomes of maternal mental course training?

## 2 Methodology

The review was conducted according to Joanna Briggs Institute of Scoping Review Methodology (22). The protocol was developed and registered with the Open Science Framework (OFS) (https://doi.org/10.17605/OSF.IO/RKGMD). The reporting was according to the Preferred Reporting Items for Systematic Reviews and Meta-Analyses (PRISMA-ScR) checklist (23), thus in line with the PRISMA scoping review extension.

### 2.1 Inclusion and exclusion criteria

The review started by identifying the inclusion criteria guided by the PCC (Population, Concept and Context) (Table 1).

**Table 1:** PCC and inclusion and exclusion criteria.

Characteristics	Inclusion criteria	Exclusion criteria
Population provided the course	Healthcare professionals; Healthcare providers; nurses; Doctors; mental healthcare professionals, Allied healthcare professionals	Healthcare professionals’ students; non healthcare professionals or providers
Population receiving the courses	Pregnant women/mothers, healthcare professionals’ primary health care, community workers, allied health providers	Women with no pregnant, non-healthcare professionals or providers; healthcare professionals’ students
Concept	Short maternal mental health, mental health of mothers, perinatal mental health courses with synonyms such as short learning programme, skills programme, short course, credit bearing or non-credit bearing short course training, course, programs, Continuous Professional Development (CPD), clinical conferences, on-job training, workshops, seminars, coaching, mentorship. In the context of the review, short courses have been defined as any additional maternal mental health course delivered in a structured manner from two hours to months with less than 120 credit hours during the perinatal period. According to World Health Organisation (3), maternal mental health conditions refer to emotional, psychological, and mental health challenges that women can experience during pregnancy and through the first year after childbirth (the perinatal period).	Any maternal mental health course with 120 credits and above which results in a professional qualification registerable with a health regulatory body.
Context	Low- and middle-income countries as classified by world bank. As reported by the World Bank (24), Low Middle-Income Countries (LMICs) account for 85% of the global population and are divided into low-income (GNI of <= $1,135), lower-middle-income (GNI$1,136 to $4,495), and upper-middle-income (GNI $4,496 to $13,935) categories. Low-income countries are primarily located in regions of sub-Saharan Africa and South Asia. Middle-income countries include nations such as India, Brazil, China, and South Africa.	Articles with all countries from high income countries (GNI > $13,935
Type of evidences	Quantitative, qualitative and mixed methods study designs; Grey literature sources in the form of academic theses and dissertations; World Health Organization (WHO); Professional organizations website	Review articles including: systematic, meta-analysis, Integrative and scoping reviews. The review studies were used to identify primary studies, provide the contextual background information and identification of primary studies as well as to discuss the results.
Language	English language articles	Articles written in other languages other than English

### 2.2 Search strategy

Search terms were derived from the combination of PCC. Two reviewers (GC; EM) implemented a three-step search strategy during the month of November and December 2025. First, they searched two relevant databases namely MEDLINE (PubMed or Ovid) and CINAHL through EBSCO Host, as they were suitable for providing evidence on short maternal mental health courses. Next, text words found in the titles and abstracts of the retrieved papers, as well as the index terms used to describe the articles were analyzed to refine the search terms.

A second search was done using all identified keywords and index terms in all the remaining data bases namely Web of Science, Scopus, African Journals online (AJOL) and Sabinet. A search for grey literature on unpublished theses and dissertations was initially carried out using EBSCOhost in the Open Dissertations database, followed by an examination of the ProQuest Dissertations & Theses Global (PQDT Global) database. The reviewers also sought information through search engines, including Google and Google Scholar, as well as the World Health Organization's professional organization website, to identify any relevant data on short maternal mental health courses. Lastly, the reference list of identified articles were searched for additional sources. The reviewers involved the librarian in designing and refining the search. A sample search from PubMed is attached in Table 2.

**Table 2:** Sample search strategy from pubMed data base.

Search words used	Number of articles retrieved
Search: ((Maternal mental health courses) AND (Healthcare providers))	
(“maternally"[All Fields] OR “maternities"[All Fields] OR “maternity"[All Fields] OR “mothers"[MeSH Terms] OR “mothers"[All Fields] OR “maternal"[All Fields]) AND (“mental health"[MeSH Terms] OR (“mental"[All Fields] AND “health"[All Fields]) OR “mental health"[All Fields]) AND (“course"[All Fields] OR “course s"[All Fields] OR “courses"[All Fields]) AND (“health personnel"[MeSH Terms] OR (“health"[All Fields] AND “personnel"[All Fields]) OR “health personnel"[All Fields] OR (“healthcare"[All Fields] AND “providers"[All Fields]) OR “healthcare providers"[All Fields]) AND (“developing countries"[MeSH Terms] OR (“developing"[All Fields] AND “countries"[All Fields]) OR “developing countries"[All Fields] OR (“low"[All Fields] AND “middle"[All Fields] AND “income"[All Fields] AND “countries"[All Fields]) OR “low and middle income countries"[All Fields]) **Translations** **Maternal:** “maternally"[All Fields] OR “maternities"[All Fields] OR “maternity"[All Fields] OR “mothers"[MeSH Terms] OR “mothers"[All Fields] OR “maternal"[All Fields] **mental health:** “mental health"[MeSH Terms] OR (“mental"[All Fields] AND “health"[All Fields]) OR “mental health"[All Fields] **courses:** “course"[All Fields] OR “course's"[All Fields] OR “courses"[All Fields] **Healthcare providers:** “health personnel"[MeSH Terms] OR (“health"[All Fields] AND “personnel"[All Fields]) OR “health personnel"[All Fields] OR (“healthcare"[All Fields] AND “providers"[All Fields]) OR “healthcare providers"[All Fields] **Low- and middle-income countries:** “developing countries"[MeSH Terms] OR (“developing"[All Fields] AND “countries"[All Fields]) OR “developing countries"[All Fields] OR (“low"[All Fields] AND “middle"[All Fields] AND “income"[All Fields] AND “countries"[All Fields]) OR “low- and middle-income countries"[All Fields]	73

### 2.3 Source of evidence selection

The process of source selection was done in two stages during January and February 2026. Prior to selection of studies, 394 duplicates were removed to remain with 195 for title and abstract screening. The first stage was based on title and abstract examination using the PCC inclusion criteria, resulting in exclusion of 84 articles based on wrong population (*n* = 23), concept (*n* = 31) and context (*n* = 30) with 111 papers deemed eligible for full text screening. Three full text papers could not be found hence second level full text screening was done on 108 papers and 93 papers were excluded based on unfinished research in the form of protocols (*n* = 3), wrong concept (*n* = 61), unclear outcomes (*n* = 6) and wrong context (*n* = 23). All selection stages were carried out by two reviewers (GC; EM) independently, and disagreements were resolved through consensus with the involvement of a third and fourth reviewer (CRO; CTM). A flowchart of the review process following PRISMA-ScR is available as Figure 1. This flowchart details the articles identified in databases, the removal of duplicates, and the screening of titles, abstracts, and full texts. The Endnote and Ryann software was used for the management of the search process.

**Figure 1:**
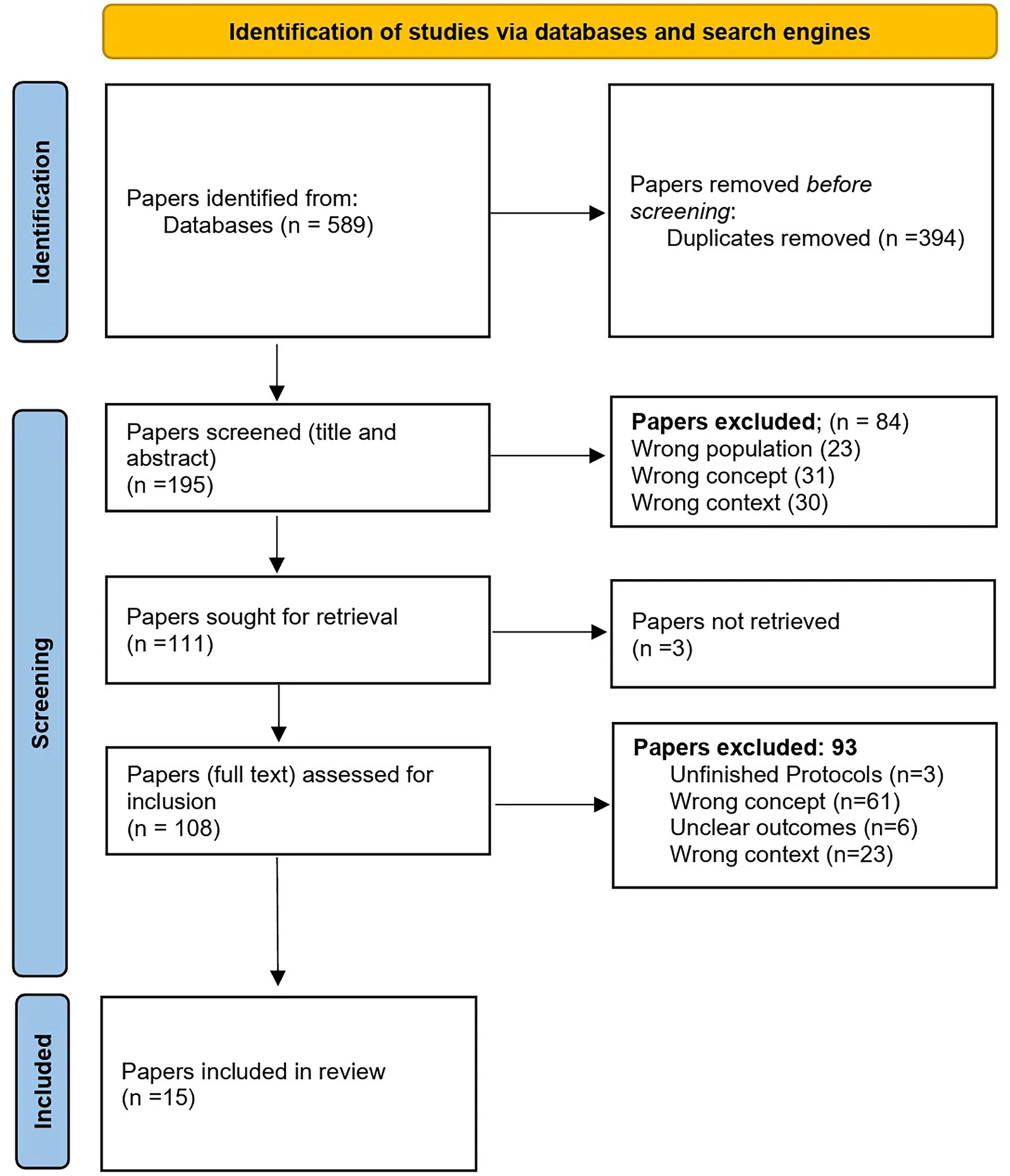
Prisma diagram showing the detailed selection of articles.

### 2.4 Data extraction process and analysis

The data extraction and analysis took place from March 2026 to May 2026. Two reviewers (MR; WH) developed a logical and descriptive summary of the results that aligned with the specific review questions was presented in the form of charting table and double checked by others (GC; CRO). The key information recorded included publication characteristics, characteristics of mental health care courses being taught, evaluation methods and outcomes (Tables 3–5). Evidence was analyzed by two reviewers (SSM; CM) through frequent counting and narrative summary followed.

**Table 3:** Publication characteristics.

Author, Year	Country	Low or Middle income	Research Design	Setting
Adefolarin et al. (25)	Nigeriaa	Middle Income	Quasi-experimental study (Two-group prestest-posttest)	Primary Health Centres (PHCs) in the Ibadan metropolis, Oyo state, Nigeria
Jannah et al. (26)	Indonesia	Middle income	quantitative quasi-experimental design, specifically described as a pre-test and post-test with a control group design	Griya Terapi Holistic Ungaran, Indonesia
Surjaningrum et al. (27)	Indonesia	Middle income	Delphi Method/Delphi Technique (Exploratory Research)	The study was focused on developing materials for posyandu cadres (community health workers) in Surabaya, Indonesia. The expert panel included practitioners working in primary health care facilities called puskesmas in Surabaya
Li et al. (28)	China	Middle income	Retrospective cohort design	Rural setting. Patient records were utilized from the Third Affiliated Hospital of Xinxiang Medical University. The study population consisted of women holding a “rural hukou” (rural household registration)
Surjaningrum et al. (29)	Indonesia	Middle income	Delphi study (Delphi method/Delphi technique); described as exploratory research	local primary health care centres (puskesmas) in Surabaya
Ventevogel & Kortmann, (30)	Afghanistan	Low income	Descriptive/Program Development Report/Protocol for a Feasibility Assessment Study	Basic health facilities in rural areas
Bhamani et al. (31)	Pakistan	Middle income	multi-phase, formative, qualitative study with expert validation. Three-phase formative study: Needs assessment (literature review + stakeholder interviews); Intervention development (based on findings); Expert validation (Content Validity Index + qualitative feedback)	perinatal mental health of pregnant women in Pakistan
Honikman et al. (32)	South Africa	Middle income	pre-post-study design	Western Cape Department of Social Development CPD programme and Stellenbosch University (midwifery students
Baumgartner et al. (33)	Ghana	Middle income	Cluster-randomized controlled trial	Rural Northern Ghana; delivered within existing women's groups (C-PrES groups) as part of the Rural Emergency Health Service and Transport (REST II) project
Smith, (34)	South Africa	Middle income	Doctoral Project; involved the development of a cinematic training package	low-resource, maternity settings
Lasater et al. (35)	Mali	Low income	Qualitative Study	Rural health centers in Sélingué district and urban facilities in Bamako
Zuccolo et al. (36)	Brazil	Middle income	Randomized controlled clinical trial	entirely online randomized controlled clinical trial (RCT) testing a digital mental health intervention package on pregnant women with depression
Poreddi et al. (37)	India	Middle income	Experimental design	Primary health centres and subcentres of selected districts, Karnataka, South India
Cui et al. (38)	South Africa	Middle income	Critical appraisal/Evaluation of a screening program	based in peri-urban Western Cape Town at the Mowbray Maternal Hospital. The program is integrated at the primary health care level, as part of the Midwife Obstetric Unit
Honikman et al. (39)	South Africa	Middle Income	Service model description + implementation monitoring (screening coverage, outcomes); Development/validation of ultra-brief screening tool; Programmatic research informing stepped-care, collaborative model	on-site, within the maternity care environment (clinic, hospital, non-governmental organisation); There is also the site at Hanover Park Midwife Obstetric Unit (MOU), Cape Town

**Table 4:** Maternal mental health course content characteristics.

Author, Year	Name of the course	Duration	Mode of delivery	Content	Course participants	Professional Groups offering course
Adefolarin et al. (25)	Training on maternal depression inclusive health education	1 day	Face to face	Culturally acceptable terms for maternal depression and addressing myths and misconceptions; The definition, symptoms, risk, and consequences of maternal depression, as well as help seeking and coping strategies; applying Health Belief Model, boosting self-efficacy; Use of IEC materials (poster, song, leaflet in Yoruba, English, Pidgin)	Primary health care workers (PHC workers), specifically non-specialist staff such as the Chief Nursing Officer (CNO), Principal Nursing Officer (PNO), Community Health Officer (CHO), and Community Health Extension Worker (CHEW)	researchers whose affiliations included the Department of Psychiatry and the Department of Health Promotion and Education, Faculty Public Health at the University of Ibadan, Nigeria.
Jannah et al. (26)	Hypnocomfort Pregnancy Multimodal Psychoeducation Media (This is the name of the specific intervention/media used)	not specified	Hybrid; as the intervention involved respondents attending a structured training session at a specific physical location, where the content was delivered using multimodal psychoeducational media comprised of digital-based materials like educational videos, relaxation audio, and digital modules	Importance of mental health during pregnancy; the impact of anxiety; and stress management techniques. Practical techniques taught included deep breathing, visualization techniques, mindfulness, and anxiety management strategies	Researchers; Pregnant women	The researchers affiliated with the Master of Psychology Program, University Semarang, Indonesia
Surjaningrum et al. (27)	The detection of mental disorders in pregnant and breastfeeding mothers	3 weeks	Face to face	Definition, causes, criteria, and incidence of mental disorders in postpartum mothers; Sharing session from those who have experienced postpartum depression; Early symptoms of depression in postpartum mothers and postpartum depression screening tools; Early intervention for postpartum depression (psychological first aid); Prevention of postpartum depression	Healthcare professionals; Posyandu cadres (community members who run posyandu voluntarily)	Psychologist, Psychiatrist, General Practitioner, Midwife, Nurse, and Survivors (of postpartum depression)
Li et al. (28)	Holistic perinatal education program	Workshops: 2–3 h (atleast1 session during pregnancy) Counselling: 30–60 min/session (≈1 per trimester)	Hybrid	labour processes, pain management techniques, breathing exercises, and prenatal and postnatal care; individualized birth plans, mental health concerns; pregnancy-related danger signs, the necessity of skilled birth attendance, and the steps involved in Birth Preparedness and Complication Readiness (BPCR)	Rural primiparous women	Experienced obstetricians. Maternal health nurses. Experienced obstetric nurses (helming the counselling sessions). Trained maternal health professionals
Surjaningrum et al. (29)	modules and training curricula for the detection of depression in pregnant and breastfeeding mothers	3 weeks	Hybrid	Definition, causes, criteria, and incidence of mental disorders (or maternal depression) in postpartum mothers. Early symptoms of depression and postpartum depression screening tools. Early intervention for postpartum depression (specifically psychological first aid, PFA). A sharing session from those who have experienced postpartum depression. Prevention of postpartum depression	Posyandu cadres (community health workers) in Surabaya	Psychologist, Psychiatrist, General practitioner, Midwife, Nurse, Survivors
Ventevogel & Kortmann, (30)	Basic mental health training modules/Mental health training modules for health care workers	8 weeks	Face to face	Schizophrenia, Major depression, anxiety disorders (including post-traumatic stress disorders), Epilepsy, Mental retardation, and Substance abuse. For nurses and midwives, the content included basic principles of non-pharmacological mental health care management, empathic listening skills, and counselling techniques	Doctors; Nurses and midwives; and Village health volunteers and traditional birth attendants	Dutch non-governmental organization HealthNet International, with local support from the Afghan Ministry of Health
Bhamani et al. (31)	Safe Motherhood-Accessible Resilience Training (SM-ART) Intervention	6 weeks 1 session/week, 60–90 min each Total ≈ 8.5 h	Face to face	Finding the Purpose of life, Dealing with Emotions, Believing in Yourself, Adapting an Optimistic Approach, Strengthening Support Systems and Relationships, Internalizing Spirituality and Humanity	Pregnant women	Facilitators, midwives
Honikman et al. (32)	Maternal Mental Health: A Guide for Health and Social Workers	Not specified (self-directed learning, enabling participants to study at flexible times and at their own pace)	Online, distance learning	Introduction to maternal mental health; Identifying maternal mental illness; Making referrals for maternal mental illness; How to help mothers with mental health problem; HIV, substance misuse, suicide, postnatal psychosis, and perinatal loss in relation to mental health issues; poverty, teen pregnancy, refugees, maternal and child abuse in relation to mental health issues	Social workers/social work assistants; Nurses; Basic and advanced midwifery students; Other registered health or social worker students and professionals, including doctors and a psychologist	the Department of Psychiatry and Mental Health and School of Child and Adolescent Health at the University of Cape Town; the Faculty of Medicine and Health Sciences and Department of Nursing and Midwifery at Stellenbosch University; the University of Waterloo; and the University of Ottawa.
Baumgartner et al. (33)	The Integrated Mothers and Babies Course and Early Childhood Development (iMBC/ECD) program	7 months	Face to face	Based on cognitive-behavioural therapy & attachment theory; Stress management, coping skills, mood regulation; Early stimulation & bonding; support child development; Key ECD messages: breastfeeding, nutrition, timely care for sick child; Communication skills with family (husband, mother-in-law)	Pregnant women and mothers of children under age 2m	Delivered by lay counsellors (referred to as ‘model mothers’) who were women from the local communities. These model mothers were supervised by staff from the Ghana Health Service (GHS) community health officers and Catholic Relief Services (CRS) REST II field staff
Smith, (34)	Cinematic Training Package	Not specified	Online	Training package on empathy in perinatal mental health promotion; Film introducing methods to integrate mental health into routine maternity care; Addresses challenges faced by maternity care workers; On-screen text highlights empathic skills; Designed for low-resource maternity settings	Maternity care workers in low-resource settings	involved interdisciplinary stakeholder collaboration and included collaboration with the Perinatal Mental Health Project (PMHP) team in South Africa
Lasater et al. (35)	Basic courses in psychiatry/Model courses	15 days	Face to face	Aimed to increase mental health competency and capacity; Enable participants to conduct mental health screening and provide initial psychosocial care	Midwives, general medicine physicians, and ANC providers	Mental health specialists located in Bamako, Mali
Zuccolo et al. (36)	Motherly app + brief online CBT	8 weeks	hybrid	Based on Behavioral Activation (BA) & CBT App modules include: Mental health (BA: activity scheduling & planning); Sleep (hygiene + relaxation); Nutrition (healthy eating, feedback, push notifications); Physical activity (step monitoring, weekly goals); Social support (in-app contact list); Prenatal support (calendar, reminders for checkups); Postnatal support (breastfeeding guidance); CBT sessions: support app use, BA practice, cognitive restructuring, relaxation, stress management	Pregnant women	Psychologists, senior doctorate level clinical psychologist, nutritionists, and app developers
Poreddi et al. (37)	Manual-based training program (or Facilitator's manual-based training program)	3 days	Face to face	An overview of Mental Disorders, Maternal Mental Health: Key Concepts, Understanding Risk Factors for Maternal Mental Disorders, Common and severe Maternal Mental Disorders, Mother-Baby Bonding (MBB), Maternal Mental Health Assessment, Role of Auxiliary Nurse Midwives in promotion and prevention of disorders, Counseling	Auxiliary Nurse Midwives	Research team from the College of Nursing and the Departments of Nursing and Psychiatry at the National Institute of Mental Health and Neuro Sciences (NIMHANS)
Cui et al. (38)	The internal training and capacity building program developed and implemented by the Perinatal Mental Health Project (PMHP)	Not Specified	Face to face	Training is focused on improving empathetic engagement styles; skills required for screening; Techniques including psycho-education, bereavement counselling, problem-solving, relaxation and breathing exercises, and couple and family counselling	Primary health workers (for counselling), Nurses and midwives (for screening), Clinical staff	The Perinatal Mental Health Project (PMHP), external clinical supervisors and peer support protocols for staff supervision, clinical services coordinator
Honikman et al. (39)	Perinatal Mental Health Project (PMHP) Stepped-Care, Collaborative Model	The counselling intervention usually takes place during pregnancy. Counselled women attend a mean of three 50-minute sessions. The counselling service is available until one year after the birth. Postnatal follow-up includes a 6–10-week postnatal telephone assessment	Face to face	Training/Capacity Building: Knowledge transfer on maternal MH; empathic engagement demos + practice/critique; reflection on staff well-being + self-care strategies. Counselling: Client-tailored; mix of couple/family therapy, MI, PST, psychoeducation, IPT, CBT; supportive counselling in some settings; scope includes social work/case management; screening for depression, anxiety, suicidality + psychosocial risk factors	Services are targeted toward women registering for antenatal care. Capacity building is provided to Maternity Care Staff of all cadres, including nurses, midwives, medical officers, and managers. Resources were extended for social workers, undergraduate medical and nursing students, workers in the non-governmental organisation sector	The counselor on site has a 4-year Bachelor of Psychology degree or a range of different qualifications depending on the organization; The PMHP is run by a small multidisciplinary team of staff with experience in public sector health and social services

**Table 5:** Evaluation methods and outcomes of maternal mental health courses.

Author, Year	Evaluation Methods	Specific Outcomes
Adefolarin et al. (25)	Intervention: One-day maternal depression training & one-month supportive supervision (6 visits). Measures: Knowledge (10-item MCQ), Skill (15-item checklist), Self-efficacy (20-point scale), Client knowledge (pre/post questionnaire). Analysis: Fisher's exact test, t-test, Poisson regression.	Training and supervision enhanced health workers’ competence and improved client knowledge and service uptake.
Jannah et al. (26)	Quantitative quasi-experimental pre-test or post-test control group design. Data analysed using univariate frequency distribution and bivariate T-test. Intervention used hypnocomfort pregnancy multimodal psychoeducational media.	Significant improvement in maternal mental health knowledge in the intervention group. Average knowledge increase. Intervention demonstrated effective enhancement of maternal mental health knowledge.
Surjaningrum et al. (27)	Delphi study using expert consensus. Evaluation methods approved: pre-test and post-test, interactive quiz, practice roleplay, and discussion with Q&A. Experts advised immediate submission of evaluation results to improve participant motivation.	Consensus established two main objectives: provide basic knowledge on postpartum maternal mental disorders and enhance cadres’ ability to detect such disorders. An additional goal identified was reducing the likelihood of baby blues symptoms in mothers.
Li et al. (28)	Retrospective cohort design using patient records. Participants divided into Intervention Group and Control Group. Outcome measures: Beck Depression Inventory-II (BDI-II) for depression, State Trait Anxiety Inventory–State (STAI-S) for anxiety, and Birth Preparedness and Complication Readiness (BPCR) knowledge and practice scores. Statistical tests included Mann–Whitney U, Wilcoxon signed-rank, independent and paired t-tests, Chi-square, and Fisher's exact tests.	Intervention Group showed significant reductions in depressive and anxiety symptoms, with marked gains in BPCR knowledge and practice scores. Higher vaginal birth rate and fewer maternal indications for caesarean delivery were observed, indicating improved maternal outcomes following the multimodal education program.
Surjaningrum et al. (29)	Delphi consensus study with expert panel evaluation of training curriculum components. Approved evaluation methods: pre-test and post-test, interactive quiz, practice roleplay, and discussion with Q&A. Experts recommended immediate submission of evaluation results to enhance participant motivation.	Consensus established key components of a training curriculum for cadres to detect maternal depression. Outcomes included agreement on two training objectives (basic knowledge of postpartum depression and improved detection ability), five training materials, six training methods, two options each for training time and duration, and six trainer qualifications.
Ventevogel & Kortmann, (30)	Participant satisfaction evaluated using a 3-item post-session questionnaire rating teaching quality, topic relevance, and difficulty. Knowledge assessed through a multiple-choice pre- and post-test.	Knowledge scores significantly increased after training. Modules on depression and psychosis were rated most valuable due to relevance and treatability in primary care. Modules on substance abuse and anxiety disorders were rated least valuable, reflecting conceptual and treatment challenges, especially regarding PTSD and substance use management in low-resource settings.
Bhamani et al. (31)	Three-phase design: Phase I (Needs Assessment): Literature review and interviews with 25 stakeholders (17 pregnant women, 8 key informants); thematic analysis used. Phase II (Development): Applied the Gunning Fog Index (GFI) across module drafts to ensure readability in single-digit scores. Phase III (Validation): Eight experts (nurses, psychiatrists, psychologists, maternal and child health specialists) evaluated modules using a 15-item checklist; Content Validity Index (CVI) calculated to assess inter-rater agreement. Two open-ended items collected qualitative feedback.	SM-ART intervention validated as feasible and high quality. Six modules achieved strong to perfect CVI values. GFI scores confirmed accessibility and readability. Expert feedback emphasized cultural relevance, interactive structure, and comprehensive facilitator guidance. The intervention is finalized for further empirical testing to evaluate its effectiveness in building resilience and addressing perinatal mental health challenges.
Honikman et al. (32)	Pre–post study design evaluating the better care Maternal Mental Health CPD book. Knowledge assessed via a 75-item multiple-choice test covering six chapters, administered before and after completion. Attitudes toward mental illness measured using the Mental Illness Clinicians’ Attitude Scale (MICA-4) pre- and post-intervention. Data analysed with the Wilcoxon signed-rank test for paired samples.	Statistically significant improvement in mental health knowledge across all six chapters, demonstrating the CPD book's effectiveness in enhancing provider understanding of maternal mental health. No significant change observed in attitudes toward mental illness.
Baumgartner et al. (33)	Quantitative data collected at baseline, post-intervention, and 8-month follow-up through CommCare-based surveys. Analyses employed linear mixed-effects models (intent-to-treat) and clustered permutation tests. Qualitative component included 33 semi-structured interviews and focus group discussions with participants, implementers, and husbands, analysed via rapid content analysis.	Outcomes evaluated at 8-month post-intervention follow-up indicated measurable improvements in maternal mental health and child socio-emotional development following the integrated, lay counsellor delivered intervention.
Smith, (34)	Included several iterative script revisions with stakeholders (PMHP counsellors, midwife) to ensure cultural accuracy and appropriate expression of distress. Conducted stakeholder feedback workshop via Zoom and collected written feedback from professionals across maternal mental health, clinical coordination, and family medicine. Administered Evaluation of Film Relevance Survey to 13 stakeholders (5-point Likert scale) and organized a group viewing session with 15 maternity care workers (midwives, nurses, and a doctor) at Hanover Park Midwife Obstetric Unit.	Stakeholders rated the cinematic training package as contextually appropriate and acceptable. 100% agreed that management approaches (empathic care, psychoeducation, health promotion, helping skills, screening, and referral) were acceptable. 92% agreed it could be integrated into existing training programs in South Africa. 85% agreed it addressed educational gaps in empathic care. Feedback led to revisions such as scene shortening, addition of on-screen text to emphasize empathic skills, and interactive discussion prompts between scenes.
Lasater et al. (35)	Methods included semi structured in-depth interviews with women (*n* = 10), midwives (*n* = 7), community health workers (*n* = 8), and mental health specialists (*n* = 4), plus focus group discussions with CHWs, auxiliary midwives, and obstetric nurses. Data were inductively coded and analysed through thematic analysis.	Integration of mental health into antenatal care (ANC) by midwives was found to be feasible and acceptable. Three main themes emerged: (1) need and acceptability of integrated mental health care among perinatal women, (2) the influence of stigma on service access, and (3) health worker support for integrating screening and psychosocial care into ANC. Midwives identified as key providers for delivering mental health screening and low-intensity psychosocial interventions. Findings suggest that group-based and talk therapy–oriented approaches align with women's existing coping practices and community structures.
Zuccolo et al. (36)	Assessments at T0 (baseline), T1 (week 4–5), T2 (posttreatment, week 8), and T3 (follow-up, 2 months postpartum). All assessments conducted by blinded assessors. Instruments include: EPDS (depression), GAD-7 (anxiety), PSS (stress), SSQS (sleep quality), SF-12 (quality of life), IPAQ-Short (physical activity), and Ryff's Psychological Well-Being Scale. App usability and user experience measured using SUS and uMARS at T2. Infant outcomes measured at T3 via the SWYC.	Change in maternal prenatal depression from T0 to T2; Change in anxiety, psychological well-being, perceived stress, depression severity, sleep quality, quality of life, and physical activity. Secondary outcomes (postnatal follow-up): Change in postnatal depression symptoms, anxiety, well-being, stress, sleep, and quality of life, plus infant developmental milestones and social/emotional outcomes at 2 months.
Poreddi et al. (37)	Participants randomly assigned to experimental (*n* = 53) and control (*n* = 57) groups. The intervention comprised 10 interactive sessions over three days, using brainstorming, case vignettes, role plays, and audio-video presentations. The facilitator's manual was developed from literature review, FGDs with ANMs, and expert input. Assessments were conducted at baseline, post-intervention, 3 months, and 6 months, using: a knowledge questionnaire, attitude assessment tool, and case vignette to test skills. Data analysis: independent t-test and repeated measures ANOVA.	The program significantly improved knowledge, attitudes, and skills related to maternal mental health. Knowledge: Experimental group's scores increased significantly post-intervention and remained higher at 3 and 6 months. Significant improvement in attitudes toward women with maternal mental disorders and professional role immediately post-intervention, though the former effect diminished by 6 months. Skills: Scores improved significantly and were maintained at 3 and 6 months. Practical impact: Experimental group identified and referred 104 women versus 39 in the control group during follow-up.
Cui et al. (38)	Mixed-methods evaluation combining secondary data review and framework-based appraisal. Program viability assessed using UK National Screening Council criteria: program condition, test validity, intervention capacity, harm–benefit balance, and implementation. Program impact analysed using peer-reviewed patient participation and self-reported outcome data and PMHP annual report data. Access to care evaluated using Gjerdingen et al. (2007)'s three-level barrier model (patient, provider, system). Financial sustainability assessed using León's (2001) “four pillars” of sustainable organizational financial management.	The program met most viability and sustainability criteria and was found financially sustainable and scalable. Reach and acceptability: 95% screening uptake (*n* = 5,407/6,347) and 90% intervention acceptance. Accessibility: >90% of counselled women reported a positive experience. Clinical outcomes: 87.8% reported symptom improvement; 91.7% positive session feedback. Postnatal follow-up (*n* = 223, 2015–2016) showed significant improvement in sadness, crying, and self-harm thoughts.
Honikman et al. (39)	Routine monitoring and evaluation system tracking service indicators: screening coverage, number of women counselled, referrals made and lost to follow-up, sessions per woman, and external agency referrals. Postnatal follow-up assessments analysed symptom score changes and problem resolution. In-depth interviews with clients and staff explored service attitudes and preferences.	Service uptake and impact: Screening coverage 71%; 22% screened required referral; 63% accepted counselling; 93% of those attended sessions, with an average of 3.4 sessions per woman. Data demonstrate effective service utilization and retention. The program informed policy, guidelines, and clinical practice, and supported creation of open-access psychoeducational and multimedia training resources.

## 3 Findings of the review

### 3.1 Publication characteristics

#### 3.1.1 Publication yearly trends

The included studies were published between 2004 and 2025, with a noticeable increase in publications in recent years. The highest number appeared in 2021 (*n* = 4), followed by 2023 (*n* = 3) and 2022 (*n* = 2). Single studies appeared in 2004, 2018, 2019, 2020, 2024 and 2025 (Figure 2).

**Figure 2:**
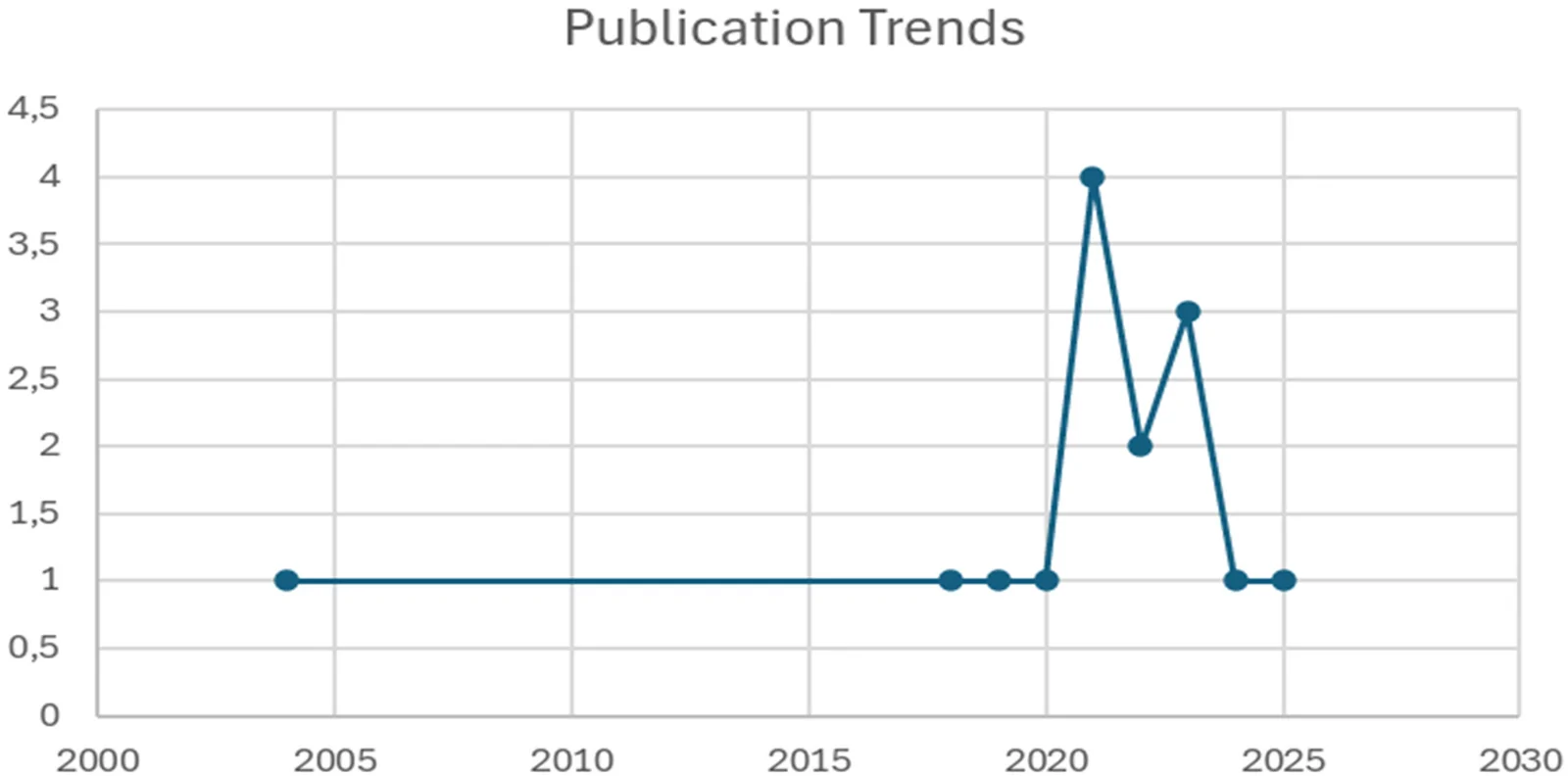
Publication yearly trends.

#### 3.1.2 Geographical distribution

According to Figure 3, the studies included in the review were conducted across ten countries, predominantly in middle-income settings (*n* = 13) and a smaller number in low-income contexts (*n* = 2). The highest representation came from South Africa (*n* = 4) and Indonesia (*n* = 3), followed by Nigeria, China, Ghana, India, Pakistan, and Brazil with one study each. Among low-income countries, Afghanistan and Mali each contributed one study.

**Figure 3:**
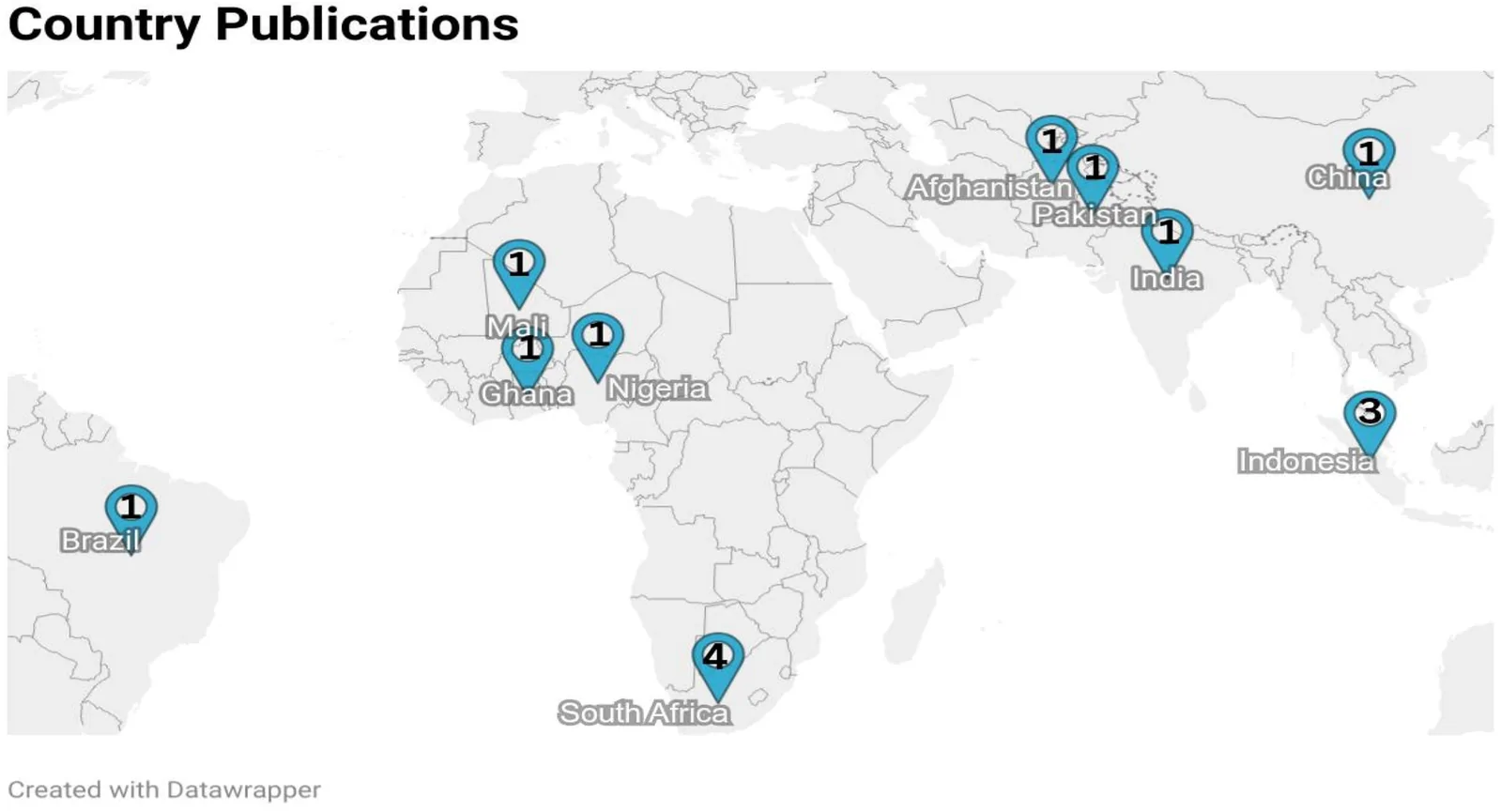
Geographical country publications.

#### 3.1.3 Research design

The included studies employed a wide range of research designs (Figure 4). Experimental and quasi-experimental designs were the most common, featuring pre- and post-test evaluations or controlled trials aimed at assessing training impact (*n* = 4). Two studies utilized the Delphi method for expert consensus in course or material development, while others adopted qualitative (*n* = 2) or multi-phase formative designs (*n* = 1) that combined needs assessment, intervention development, and expert validation. This classification facilitated a clearer understanding of the methodological diversity across studies. Additional methodologies included a retrospective cohort study, program development report, doctoral project, critical appraisal, and service model evaluations, each contributing distinct perspectives on training feasibility, implementation, and effectiveness.

**Figure 4:**
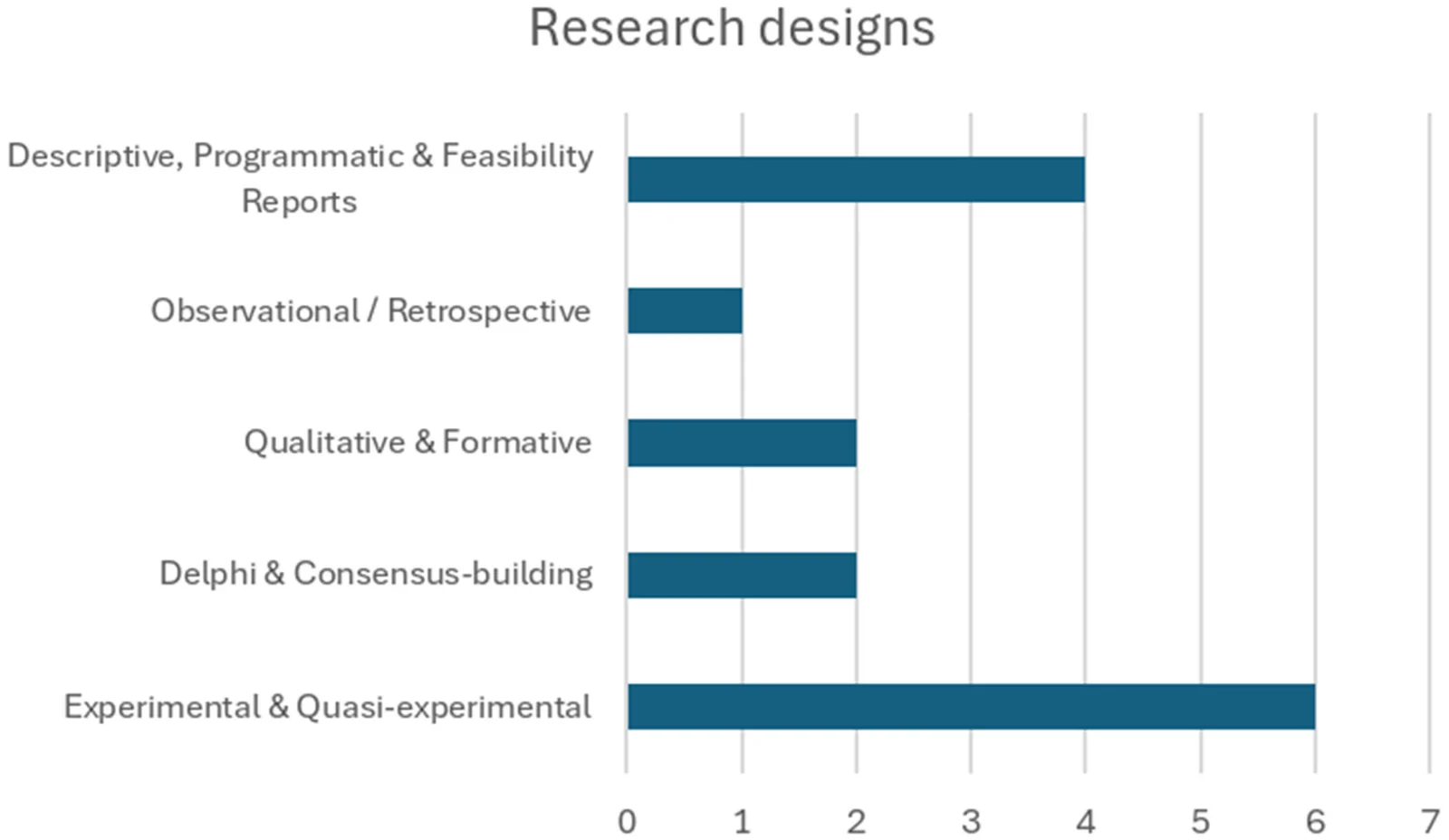
Research designs from the identified evidences.

#### 3.1.4 Setting

The fifteen studies were conducted across a range of clinical, community, academic, and digital environments, reflecting the varied contexts in which maternal mental health training is delivered (Figure 5). The majority were situated in primary health care settings (*n* = 8), including primary health centers, subcenters, and community-based clinics in countries such as Nigeria, India, Indonesia, and South Africa. Community based and rural settings accounted for four studies, encompassing women's groups and rural health facilities in Ghana, China, Mali, Pakistan, and Afghanistan, where access to mental health care is limited. Academic or institutional environments (e.g., universities and hospitals) featured in two studies, such as the Third Affiliated Hospital of Xinxiang Medical University and Stellenbosch University's CPD programme. Finally, one study was conducted entirely online, evaluating a smartphone assisted cognitive behavioral intervention for pregnant women in Brazil.

**Figure 5:**
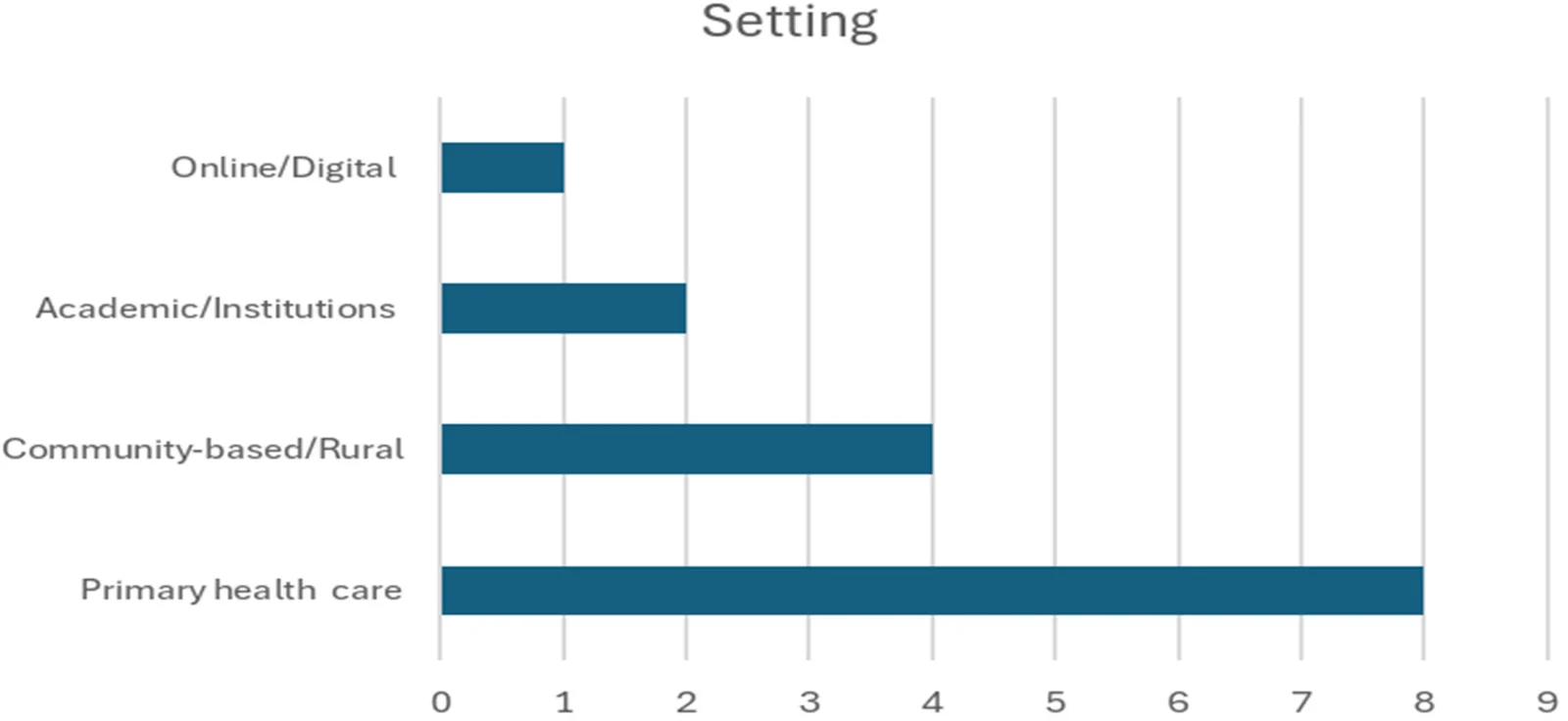
Setting in which maternal mental health courses were delivered.

#### 3.1.5 Duration

Among the fifteen studies, eleven explicitly reported duration, while the remainder (*n* = 4) did not specify exact timeframes. The mental health courses were categorized based on timing, intensity, and duration. Short-term is related to antenatal, intrapartum and 4 to 6 weeks postpartum while medium term varies from 6 weeks up to 6 months postpartum with the aim of promoting maternal recovery. Long-Term Interventions looks beyond 6 months to 1 year postpartum and through subsequent pregnancies. Based on these operationalizations, for this review, short-term programs were the most common, with durations ranging from a single-day session (*n* = 1) to multi-day or short course formats lasting between 3 days and 3 weeks (*n* = 4). Medium term interventions lasted 6–8 weeks (*n* = 3). Two programs were classified as long-term, including one that extended over approximately seven months and another that combined a single workshop with trimester-based counseling sessions spanning the entire pregnancy period. In some cases, such as the Perinatal Mental Health Project in South Africa, the intervention was ongoing throughout pregnancy, averaging three 50-minute counseling sessions with continued postnatal follow-up up to one year. Additionally, one study described a self-paced or flexible learning format (Figure 6).

**Figure 6:**
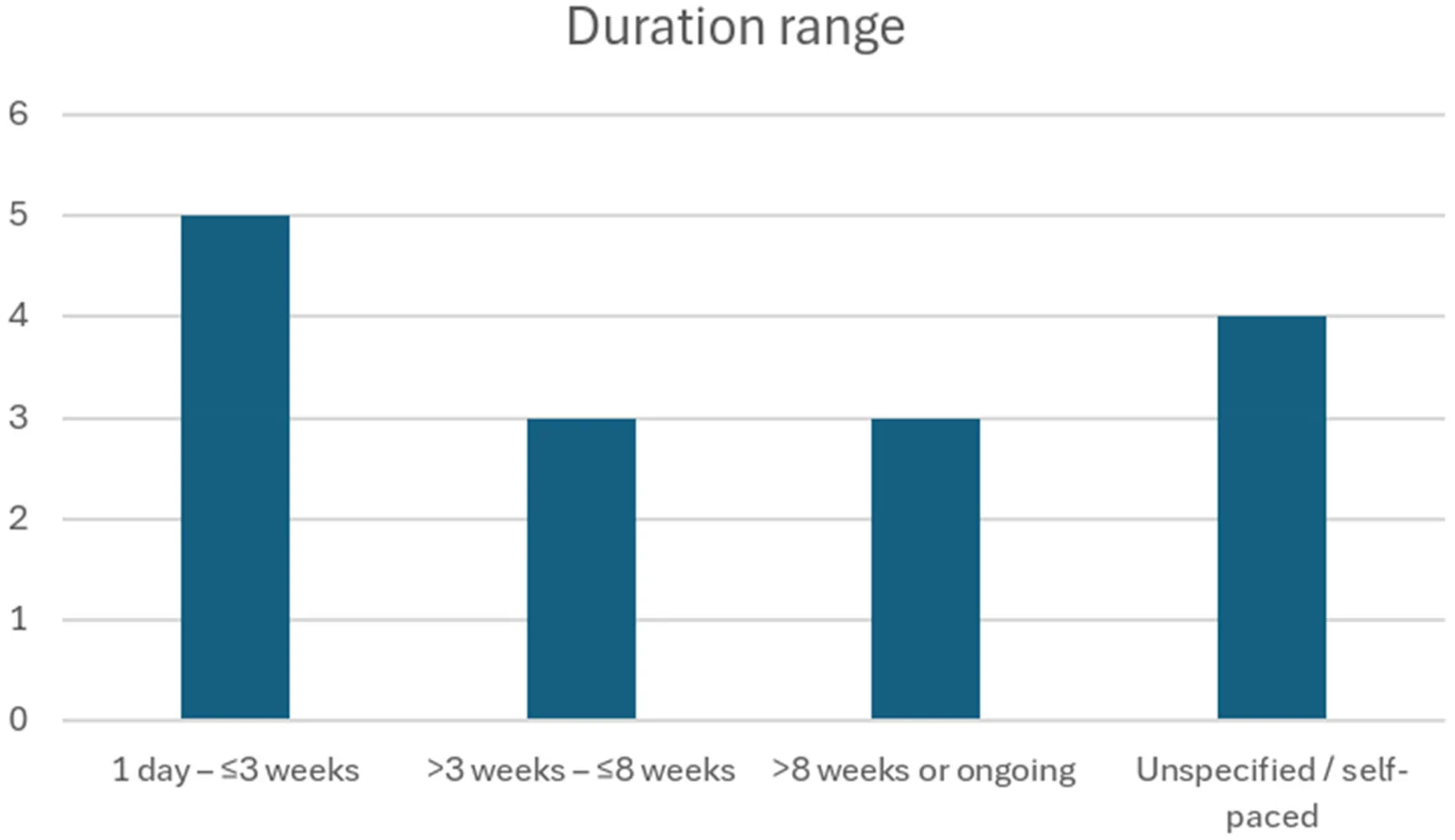
Duration of the maternal mental health course.

#### 3.1.6 Recipients of short courses on maternal mental health

A majority of the Interventions (*n* = 8) were aimed at healthcare professionals such as primary healthcare workers, nurses, midwives, social workers, medical officers. Along with community-based providers (*n* = 3), including community health volunteers. Conversely, six studies were designed for pregnant women or new mothers, either as direct recipients of mental health education or as participants in psychosocial interventions. From these, two of the studies involved mixed participants, incorporating both trainees (health and social care workers) and clients (pregnant women) (Figure 7).

**Figure 7:**
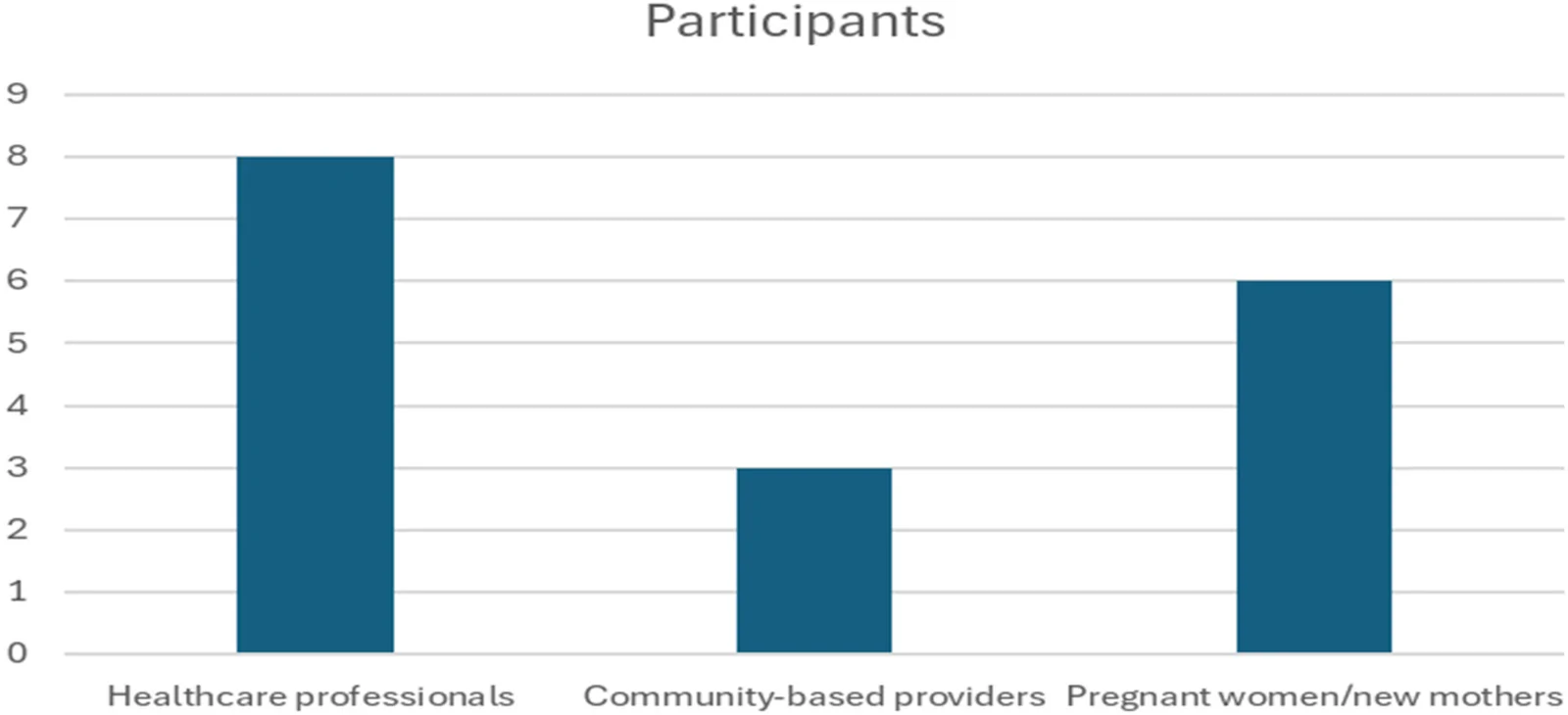
Participants involved in the maternal mental health course**(s).**

#### 3.1.7 Professional groups offering the short courses on maternal mental health

The majority of initiatives (*n* = 7) involved clinical and healthcare professionals, including psychiatrists, psychologists, obstetricians, midwives, nurses, counselors and general practitioners. Many of whom collaborated with survivors of postpartum depression or other lived experience advocates. A second major group (*n* = 4) were university or research led, often originating from departments of psychiatry, psychology, public health, or nursing. Non-governmental and international organizations featured in three studies. While community-level facilitators, such as trained lay counselors or “model mothers,” accounted for one study (Figure 8).

**Figure 8:**
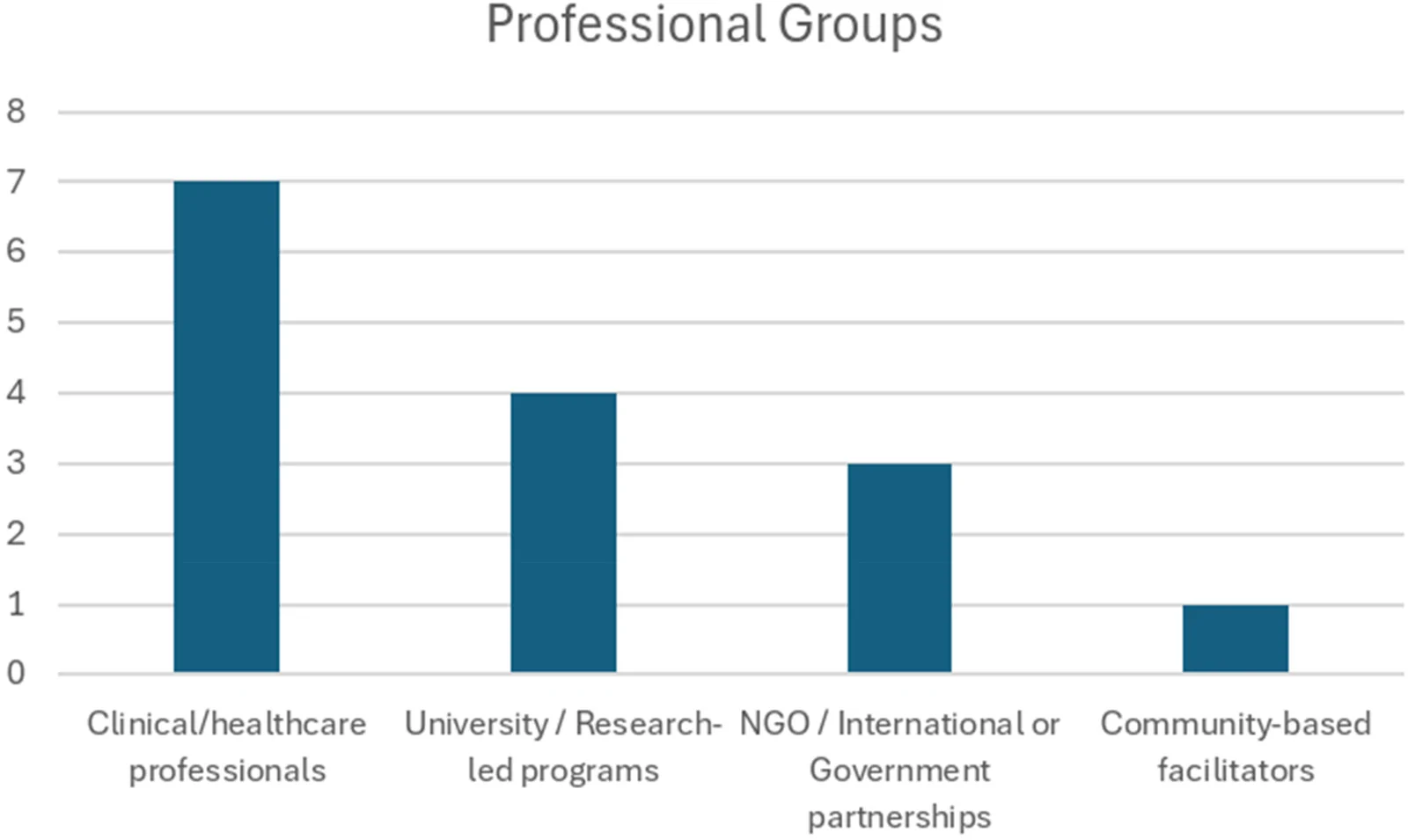
Professional groups involved in delivering the maternal mental health course.

##### 3.1.7.1 Mode of delivery

A total of nine studies implemented the courses face to face, typically through workshops, counseling sessions, or group-based training in clinical or community settings. Hybrid delivery models, which combined physical sessions with digital or multimedia components, were used in four studies. Finally, fully online or distance-learning modes were adopted in two studies (Figure 9).

**Figure 9:**
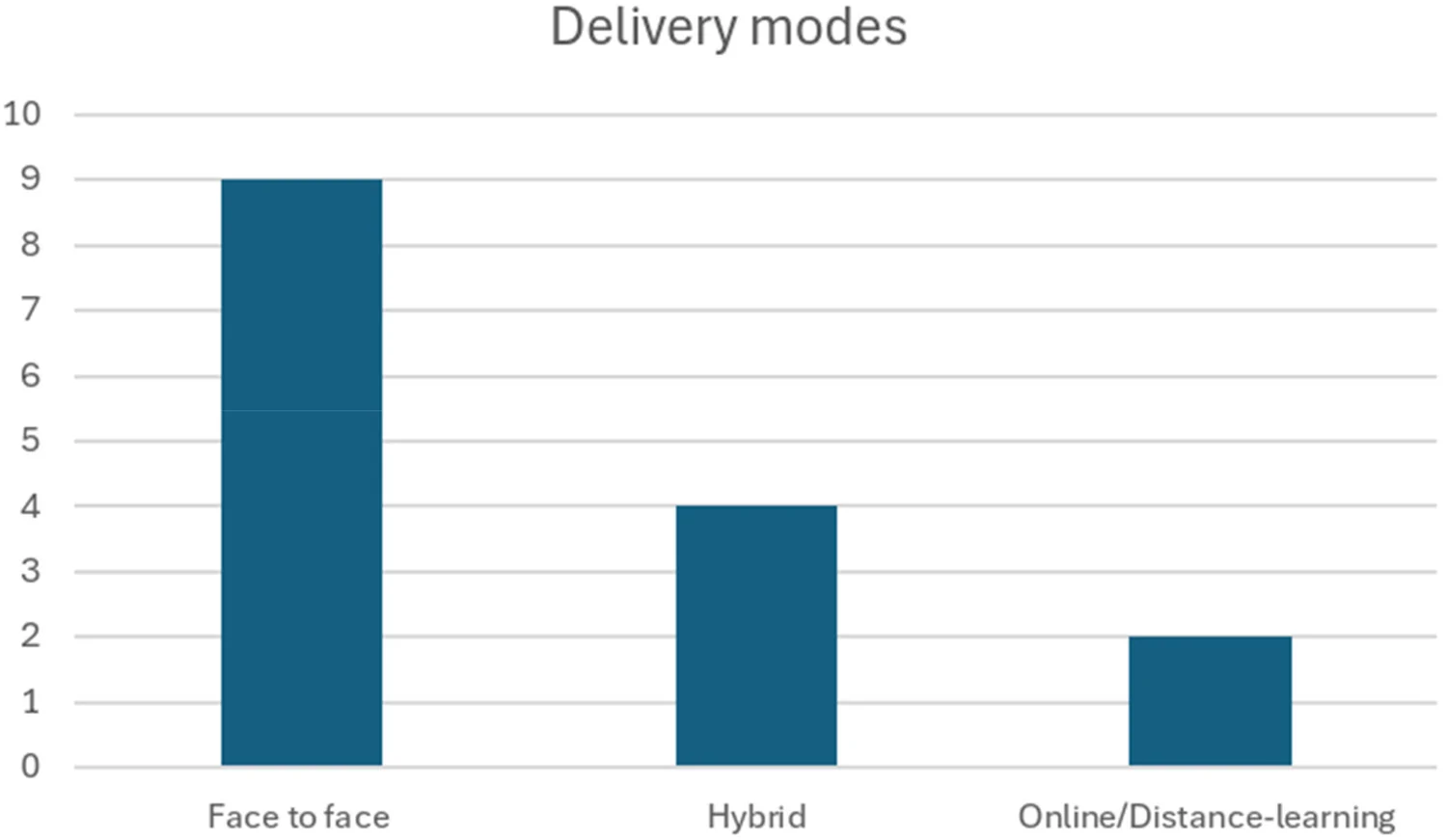
Delivery modes for maternal mental health courses.

### 3.2 Content of maternal mental health courses

The content of the maternal mental health courses across the 15 included studies demonstrated substantial thematic diversity, reflecting different priorities in prevention, clinical skill building, and psychosocial support. Despite this variation, most programs shared a central goal: to strengthen awareness, detection, and management of maternal mental health challenges, particularly depression and anxiety, during the perinatal period. To systematically describe this diversity, course content was categorized into five major thematic clusters based on learning focus and training objectives (Figure 10):

1. ***Clinical Knowledge and Screening Skills (n*** ***=*** ***5*)**; Courses in this category provided foundational or advanced knowledge on maternal mental health disorders, diagnostic criteria, and screening tools. They emphasized early detection, use of validated instruments (e.g., for postpartum depression), and mental health assessment in routine maternal care.
2. ***Counseling and Psychosocial Support Techniques (n*** ***=*** ***5)***: This category included courses designed to build practical counseling, empathy, and communication skills for frontline providers. Content covered psychoeducation, supportive listening, bereavement and problem-solving counseling, family-based therapy approaches, and stress reduction techniques. Notably, the PMHP programs in South Africa and the cinematic empathy training for maternity staff emphasized empathic engagement, while the Afghanistan training module (28) strengthened basic counseling and empathic care skills among nurses and midwives.
3. ***Stress Management, Emotional Resilience, and Self-Efficacy (n*** ***=*** ***3)***: Interventions under this category focused on mental wellness, coping strategies, and personal empowerment for pregnant women. These included mindfulness, relaxation, cognitive restructuring, deep breathing, visualization, and self-reflection exercises.
4. ***Integrated Maternal and Child Health Frameworks (n*** ***=*** ***2)***: A smaller subset of programs combined maternal mental health education with broader maternal and child health goals, such as birth preparedness, early childhood development (ECD), and nutrition. The Ghanaian lay counselor program and the rural primiparous women's multimodal perinatal education study are examples, linking emotional well-being with practical health outcomes.

**Figure 10:**
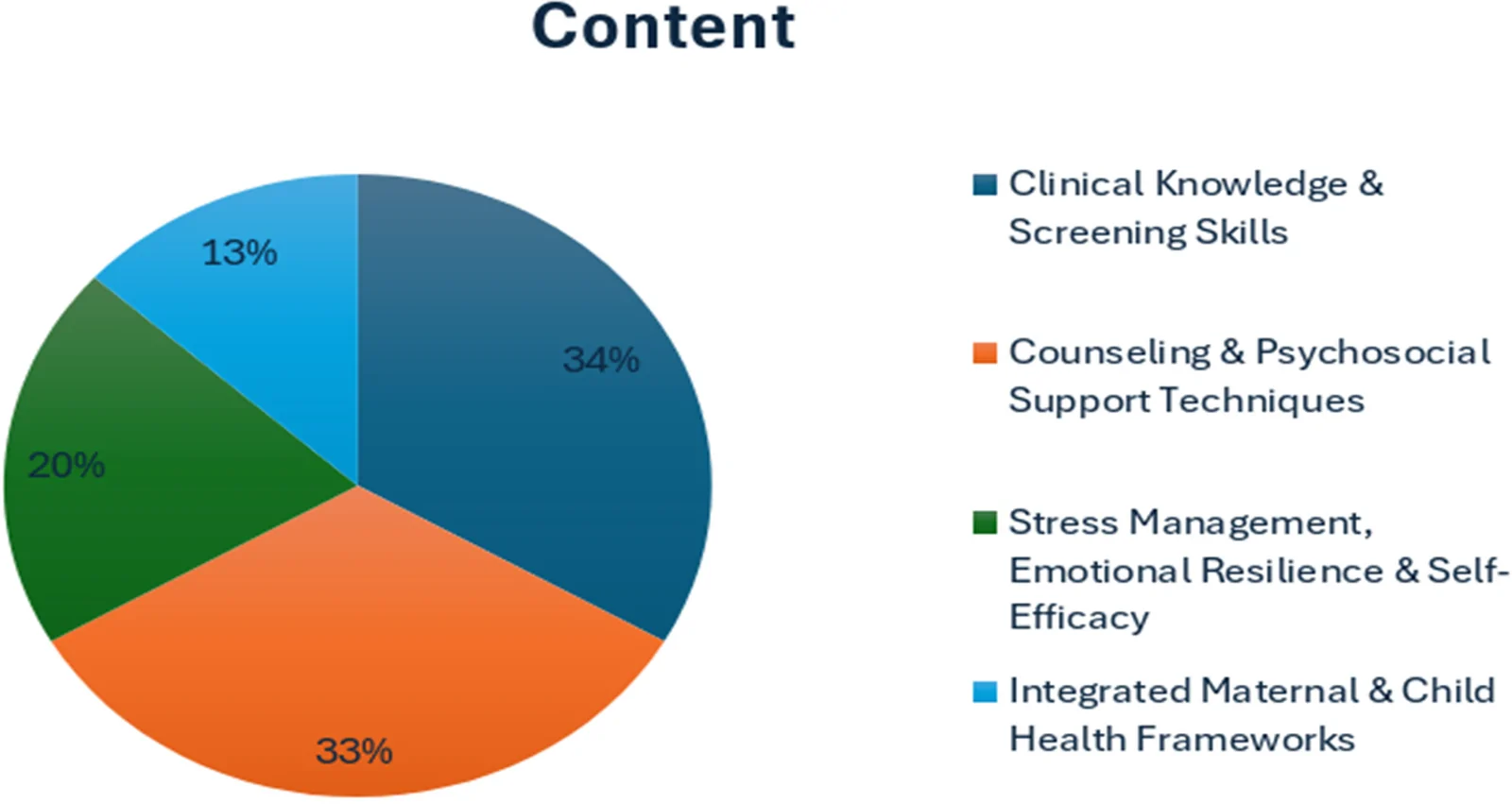
Content of maternal mental health courses.

### 3.3 Evaluation methods and outcomes

The evaluation methods used across the fifteen studies reflected a strong emphasis on assessing knowledge and program feasibility, with fewer studies measuring long-term clinical outcomes. Nearly half (*n* = 7) of the programs used quantitative pre–posttests to evaluate improvements in participant knowledge, skills, or self-efficacy, often through multiple choice questions or structured checklists. While 3 programs incorporated attitude or behavior measures, using standardized scales such as the Mental Illness Clinicians’ Attitude Scale (MICA-4) or observed role plays to gauge shifts in professional empathy and confidence (Table 5).

Clinical outcome evaluations were reported in 4 studies, measuring participant or patient-level indicators such as depression (EPDS, BDI-II), anxiety (STAI-S, GAD-7), and maternal well-being across baseline and follow-up periods. Over half (*n* = 8) conducted process and program evaluations, assessing the acceptability, feasibility, or sustainability of training interventions. These included Delphi consensus exercises, stakeholder workshops, routine service monitoring, and mixed-methods analyses of program viability and access barriers (Table 5).

Evaluation approaches across the included studies mainly focused on changes in participants’ knowledge and skills, with fewer assessing behaviour change, clinical impact, or system-level outcomes. Most studies (*n* = 9) reported significant improvements in participants’ knowledge, competence, or self-efficacy following the training. A smaller number (*n* = 4) evaluated behavioral outcomes, such as improved screening, referral practices, and communication with clients. Only three studies assessed clinical or client-level outcomes, showing reductions in depressive and anxiety symptoms and improvements in maternal and child wellbeing. Finally, three programs evaluated system-level outcomes, which demonstrated strong integration within public health systems and sustained service utilization. Overall, most evaluations concentrated on learning outcomes, with fewer extending to practice change or broader health and system effects (Table 5).

No study reported adverse or harmful effects; however, several noted limited or mixed findings. One program showed no significant change in attitudes despite knowledge gains. While another observed that attitude improvements diminished after six months. Additionally, one early program development study found lower perceived relevance of certain modules (e.g., anxiety and substance use), highlighting content adaptation challenges in low-resource settings (Table 5).

## 4 Discussion

### 4.1 Publication characteristics

Marked regional and income level disparities define the current landscape of maternal mental health (MMH) courses offered within health professions education (HPE) across WHO regions. While there is growing momentum to integrate MMH content into training frameworks, progress remains uneven, with activity concentrated in middle-income settings and significant gaps persisting in low-income contexts (4). The available evidence reveals a clear concentration of MMH related educational programs in middle-income countries (MICs), notably in the African, South East Asian, and Western Pacific regions (40, 41). These countries benefit from stronger institutional capacity, government and academic collaboration, and donor support that facilitate program development and evaluation. However, this could also indicate lack of evidence dissemination in low-income countries hence the need for capacity building especially to the early researchers (32, 41).

Pioneering initiatives such as the Perinatal Mental Health Project (PMHP) in South Africa and the Thinking Healthy Programme (THP) in Pakistan have demonstrated scalable, task-shared training for non-specialist health workers within maternal and child health systems (32, 40). Similarly, capacity-building interventions in Pakistan, Indonesia, Nigeria, Ghana, and Brazil have integrated MMH modules into pre-service and in-service programs for nurses, midwives, and primary care providers, often using short, competency-based workshops and hybrid delivery models (42, 43).

By contrast, in low-income settings, MMH education remains largely absent from formal curricula, with limited examples of structured training for health professionals (35). Where interventions exist, such as in Mali, they are often small scale pilots embedded within broader maternal and child health programs rather than standalone educational initiatives while workforce shortages and limited funding impede further scaling (44, 45). The lack of national frameworks and training resources in these contexts reflects systemic barriers to workforce development and health education reform. Overall, regional and contextual variation in MMH courses for health professions education mirrors global inequities in health education infrastructure. Middle-income countries are driving innovation in curriculum integration and task sharing, whereas low-income contexts remain largely unrepresented in formal training initiatives. There is a need to adapt evidence-based integrated MMH modules into the health program generic curricula.

Research on MMH education adopts a wide variety of experimental, quasi-experimental, and developmental designs, reflecting the efforts to identify effective and scalable training models for health professionals in both high and low resource contexts. The predominant methodologies are pre–post and quasi-experimental designs, which assess immediate gains in knowledge, confidence, and clinical skills following training interventions. For instance, Adefolarin et al. (25) conducted a quasi-experimental study in Nigeria evaluating education inclusive of maternal depression for primary healthcare workers, demonstrating significant post-training improvements in screening competence and confidence.

In low and middle income countries (LMICs), before and after comparative as well as quality improvement designs have been widely used to evaluate digital and workshop-based modules for nurses, midwives, and community health workers. These designs consistently show statistically significant learning gains and high participant satisfaction (46, 47). Studies also highlight the role of hybrid and e-learning modalities, which enable flexible participation and are particularly valuable in rural and resource limited settings (48).

Beyond outcome evaluation, qualitative, Delphi-based, and participatory curriculum development studies have played a crucial role in designing culturally relevant and context-sensitive MMH curricula. For example, Surjaningrum et al. (27) used a Delphi process in Indonesia to co-develop maternal mental disorder detection modules for community health workers, while also ensuring linguistic and cultural adaptation. Similarly, iterative consensus-building processes have been applied in developing respectful maternal care and perinatal training frameworks in Malawi and Tanzania, enhancing contextual appropriateness and stakeholder ownership (49, 50).

There is a need for a maternal mental health (MMH) education research shift to address existing evidence gaps and strengthen the inclusion of longitudinal studies, as recommended by WHO (3). Specifically, there is a need to promote a locally appropriate research agenda that prioritizes expanding implementation science to scale up integrated services and testing the effectiveness of brief psychological and psychoeducational interventions. In addition, research is needed to identify the most feasible and effective approaches for training evaluating training strategies for midwives, nurses, clinicians, doctors, and community health workers to deliver perinatal mental health services in resource-constrained settings, while also considering broader social determinants and holistic maternal wellbeing.

Most MMH initiatives in LMICs are implemented within primary health care or community based settings, where integration into existing maternal and child health services maximizes sustainability and scalability (40, 51). The Perinatal Mental Health Project in South Africa and similar task-sharing models in India, Pakistan, Ghana, and Indonesia demonstrate that embedding MMH content within PHC systems enhances provider capacity and facilitates direct translation of skills into clinical practice (52, 53). Conversely, programs affiliated with universities and hospitals, while less prevalent in LMICs, contribute significantly to postgraduate and specialist education and act as evaluation and innovation hubs for advancing MMH pedagogy (54).

Training duration and intensity vary based on the cadre and program objectives. Short-term intensive workshops (1–3 weeks) are most common across LMICs, supporting rapid upskilling while minimizing service disruption (40). Medium-term interventions, such as Pakistan's Safe Motherhood Accessible Resilience Training and Brazil's Motherly app, combine structured learning with psychosocial support and have shown promising outcomes for both provider knowledge and maternal well-being (15, 55). Longer-term models, such as Ghana's Integrated Mothers and Babies Course (iMBC/ECD), enable ongoing mentorship and supervised practice for sustained skill retention (56, 57). Together, these findings suggest that there is no single optimal training duration for maternal mental health education. Instead, training intensity should be tailored to the target workforce, learning objectives, and available resources, with ongoing mentorship and opportunities for supervised practice playing a critical role in sustaining competency beyond initial training.

Although face-to-face training remains the predominant delivery mode, valued for its cultural adaptability, interactivity, and affinity for relationship building, there is growing adoption of hybrid and online modalities to improve reach and flexibility. Programs in Indonesia and Brazil have successfully utilized blended learning and mHealth platforms, integrating digital modules, peer discussions, and guided therapist sessions to extend MMH education to remote areas (58, 59). Notably, research consistently finds that content quality, contextual relevance, and supportive supervision, rather than delivery format, most strongly predict competency acquisition and retention (60, 61). These evolving implementation models signify a pedagogical shift toward flexible, integrated MMH training ecosystems that bridge formal and community systems.

### 4.2 Maternal mental health content and competency characteristics

The content of MMH courses demonstrates a deliberate shift toward learning that is role specific and grounded in competency and thus aligning with the World Health Organization's (9) guidance for integrating mental health into maternal and child health (MCH) services. Core modules consistently emphasize foundational knowledge, including the recognition of perinatal mood and anxiety disorders, understanding risk factors, and the impacts of untreated conditions on maternal and infant health outcomes (5). Building on this foundation, MMH curricula also prioritize screening and assessment competencies, equipping providers to administer and interpret validated tools such as the Edinburgh Postnatal Depression Scale (EPDS), Patient Health Questionnaire (PHQ-9), and Generalized Anxiety Disorder Scale (GAD-7) (61). These practical assessment skills are frequently paired with psychosocial and counseling interventions, including psychoeducation, problem-solving therapy, empathic communication, and stress management, which are particularly effective and scalable for non-specialist providers in low and middle income countries (40, 41, 62).

Specialized modules extend this foundation by incorporating pharmacological management content for prescribers and nurses, emphasizing the safe use of psychotropic medications during pregnancy and lactation (63) and evidence-based psychotherapies such as Cognitive Behavioral Therapy (CBT), Interpersonal Therapy (IPT), and approaches grounded in resilience. While in some cases even adapting for delivery by trained community or lay workers in settings such as Ghana and Pakistan (40). Increasingly, MMH course design also integrates cultural and holistic dimensions, reflecting principles of care informed by trauma, maternal and infant bonding, and equity based frameworks that account for the cultural and social determinants of perinatal well-being (4). Together, these trends indicate a pedagogical evolution toward contextually grounded, skill oriented education designed to bridge the global treatment and training gap in maternal mental health.

The participant composition of maternal mental health (MMH) education programs demonstrates a focus on capacity building for healthcare providers and enhancing psychosocial literacy among women. Most MMH training initiatives prioritize frontline healthcare professionals, including nurses, midwives, and primary care physicians which are groups that form the first point of contact for perinatal women and are critical for early detection and intervention of mental health conditions (20, 25). These programs emphasize practical competencies, including screening for perinatal depression and anxiety, effective communication, and safe psychotropic medication management during pregnancy and lactation (53). Complementing this, several interventions directly involve pregnant and postpartum women as active participants in structured education and psychosocial programs so as to build sessions which are designed to improve their mental health literacy and emotional well-being (24, 38). Furthermore this workforce strengthening aligns with WHO's task-sharing strategy, which enables non-specialist providers to deliver essential psychosocial interventions within primary care systems (9, 41). In low resource contexts, programs have demonstrated success in empowering community health workers and lay counselors, for example, the Thinking Healthy intervention in Pakistan and similar community-led perinatal programs in South Africa and Nigeria, by equipping them with structured counseling skills for perinatal depression management (5, 53, 63).

Moreover MMH training delivery is often interprofessional and multi-sectoral, and involves partnerships among academic institutions, health ministries, and NGOs. These collaborations enhance implementation fidelity and sustainability by combining clinical expertise with community engagement (53). Instructors typically include psychiatrists, psychologists, midwives, and experienced maternal health educators, reflecting the cross disciplinary nature of MMH care. Community models further extend this reach by engaging lay providers and peer volunteers such as Ghana's “model mothers” and Indonesia's posyandu cadres, who help to diffuse psychosocial knowledge and support at the grassroots level (3, 41). NGOs along with partnerships between universities and governments play a central facilitative role in coordinating these training networks to ensure both quality control and contextual adaptation. Importantly, several programs also integrate the voices of women with lived experience of perinatal mental health conditions into training design or delivery so as to empathy and realism in provider education. This diversified instructional ecosystem, connecting formal health systems and community networks, enhances cultural adaptability and continuity of care while also ensuring that knowledge transfer supports long-term and a locally relevant MMH practice.

### 4.3 Evaluation and outcome insights

Evaluation of maternal mental health education reveals consistent short-term improvements in provider knowledge, skills, and self-efficacy, with emerging though limited evidence of sustained behavioral change and clinical benefit. Most studies employ quantitative pre–post designs to assess training effectiveness, using structured questionnaires, multiple-choice tests, or validated psychometric tools such as the Edinburgh Postnatal Depression Scale (EPDS) and the Generalized Anxiety Disorder-7 (GAD-7) to capture learning outcomes. These designs consistently report significant gains in knowledge of perinatal depression and anxiety, enhanced screening proficiency, and improved confidence in psychosocial counseling (25, 43, 64). More rigorous quasi-experimental and randomized trials, including those evaluating continuing professional development modules and interventions delivered by lay counselors, reinforce these findings by demonstrating measurable gains in applied clinical competence (41, 42).

Process and programmatic evaluations complement these outcome studies by examining feasibility, acceptability, and sustainability. Consensus-based and Delphi validation approaches ensure curricular relevance, while routine monitoring systems such as those used in the Perinatal Mental Health Project (PMHP) track implementation indicators including screening uptake, counseling sessions, and referral completion (65).

Qualitative interviews also highlight the importance of contextual fit and provider perceptions in sustaining engagement (64). At the client level, several interventions show meaningful clinical benefits: mothers receiving care from trained providers report significant reductions in depressive and anxiety symptoms, improved emotional well-being, and, in some cases, better birth outcomes and infant development (66, 67). Despite these encouraging trends, the evidence base remains constrained by methodological limitations underscoring the need for standardized evaluation frameworks and longitudinal impact assessments (68, 69).

### 4.4 Gaps and challenges

Despite growing recognition of maternal mental health (MMH) as a global health priority, pervasive gaps remain across research, education, and implementation domains (4, 51). From a methodological standpoint, the evidence base is limited by weak to moderate study quality, with most interventions relying on quasi-experimental or uncontrolled designs, small sample sizes, and short follow up periods (51, 70). Few comparative or longitudinal studies assess sustained competency or clinical outcomes, and most evaluations depend on self reported measures rather than objective assessment of clinical performance or patient well-being (35, 70). These methodological constraints hinder the establishment of best practices informed by evidence for MMH training and care delivery, particularly in low and middle income countries (54, 71).

Persistent curricular and competency gaps further undermine workforce preparedness. MMH content remains inconsistently integrated within pre-service medical, nursing, and midwifery curricula (66). Specialist training in perinatal psychiatry is rare globally, and few countries require exposure to psychiatric illness management in the perinatal period (72). Existing educational programs often emphasize depression and anxiety, neglecting complex conditions such as postpartum psychosis, PTSD, and obsessive-compulsive disorder, which demand specialized competencies (4, 73). Moreover, while most training improves provider knowledge and confidence, attitude change and empathy gains are less consistent and often diminish over time, revealing a lack of emphasis on sustained stigma reduction and reflective practice (34, 70).

At the systemic level, fragmented policy frameworks and limited institutional investment continue to constrain MMH integration into national health systems. Many countries lack dedicated perinatal mental health policies, treatment pathways, or funding mechanisms, resulting in inconsistent implementation and accountability (45, 74, 75). The absence of cross-sector collaboration between maternal and mental health services also impedes continuity of care (9, 72). In low-resource settings, workload pressures, staff shortages, and time constraints are major barriers to both training participation and screening implementation (13, 44). Broader mental health frameworks addressing mental, neurological, and substance use (MNS) disorders often overlook MMH competencies such as reproductive psychopharmacology, trauma-informed care, and infant-mother bonding assessment (9, 70).

Finally, stigma and equity barriers persist as major deterrents to help seeking and effective intervention. Many women avoid disclosing symptoms due to fear of judgment, discrimination, or loss of child custody (4, 70). Cross national studies show that ethnic minority and low-income women are less likely to be screened for mental health issues or offered appropriate care (73). While structural stigma within healthcare institutions and sociocultural norms around motherhood tend to reinforce these disparities (9, 70, 76).

### 4.5 Implications for health professions education and global health policy

The evidence base underscores an urgent need to institutionalize maternal mental health (MMH) as a mandatory pre-service competency within health professions education (HPE). Studies show that short-term, workshop interventions consistently improve providers’ knowledge, confidence, and self-efficacy, yet these gains are seldom sustained in the absence of structured curricular integration (77, 78). Embedding MMH within nursing, midwifery, and primary care curricula is critical to ensuring that future providers possess not only diagnostic awareness but also practical competencies in screening, psychosocial intervention, and referral coordination (79).

Pedagogically, HPE must prioritize a skills focused, experiential learning delivered through interactive and accessible modalities. Evidence from both high and low resource settings demonstrates that hybrid and e-learning are as effective as face-to-face formats when they emphasize applied learning, contextual relevance, and reflective practice (80, 81). Incorporating Interprofessional Education principles enhances collaboration and builds coordinated referral networks essential for comprehensive perinatal care (82). In parallel, MMH curricula should embed cultural humility and trauma informed care frameworks to address the inequities and stigma that hinder help seeking among mothers (83). Approaches informed by trauma within perinatal education foster practice that is empathic and nonjudgmental, and are linked to reduced secondary trauma among providers and improved maternal satisfaction (78, 81).

From a global health policy perspective, few nations possess dedicated perinatal mental health policies, and existing maternal and child health (MCH) frameworks often neglect mental health explicitly (81). Governments should embed MMH training mandates within national workforce development strategies, supported by funding for supervision, continuing education, and digital infrastructure (62). In low and middle income countries, policy frameworks should expand scalable task-sharing models to empower non-specialist providers and community health workers to deliver basic psychosocial support under professional supervision (79, 84). Ultimately, bridging the education and policy divide requires coordinated efforts among academia, professional bodies, and ministries of health to ensure that MMH competencies are standardized, culturally responsive, and sustainably integrated within both pre-service and in-service systems. Doing so will not only strengthen the global health workforce but also advance equity and quality of care for mothers and infants worldwide.

### 4.6 Future research and recommendations

Future research must move beyond documenting short-term knowledge gains to establishing an evidence based framework for the sustained integration of Maternal Mental Health (MMH) education into health professions training, particularly in LMICs. Current studies rarely measure whether improved knowledge and self-efficacy translate into long-term clinical competence or better maternal outcomes. Therefore, future studies should include longitudinal follow-up to assess whether gains in provider knowledge, confidence, and screening behavior persist over time. Incorporating behavioral and practice measures such as changes in screening frequency, referral rates, and patient engagement, will clarify real world impact. Evaluations should also include practical skill assessments (e.g., OSCEs or simulations) and test the value of refresher or booster modules to maintain skills over time.

In addition, research must compare different teaching modalities, namely face-to-face, hybrid, and fully online, to determine the most effective and scalable models for various contexts. Given the success of digital learning platforms, studies should optimize online delivery by testing interactive, multimedia, and case based methods. Comparative research should also identify who delivers MMH training most effectively (psychiatrists, nurses, midwives, or community facilitators) to inform workforce planning and sustainability. Furthermore, research should evaluate effective models for integrating MMH content into undergraduate education and ensure alignment with national and institutional competency frameworks. Curricula should expand beyond depression and anxiety to include postpartum psychosis, PTSD, and OCD, reflecting the full spectrum of perinatal mental disorders. Moreover, training interventions must be culturally and contextually adapted, especially in LMICs, to reflect local beliefs, languages, and care systems. Research should rigorously evaluate community-led approaches for their effects on stigma reduction, and patient trust. Studies must also ensure diverse participant representation, including providers serving marginalized populations, to enhance relevance and generalizability.

## 5 Conclusions

The reviewed evidence indicates a growing but geographically uneven effort to integrate maternal mental health training into health professions education, particularly within frontline and community healthcare systems in low and middle income countries. Publication trends reveal a marked increase in studies since 2020, suggesting that MMH integration into health curricula and primary care has become a recent global priority. Geographically, research activity remains concentrated in a limited number of middle-income contexts, most prominently South Africa and Indonesia, while representation from low-income countries such as Afghanistan and Mali remains minimal. This imbalance suggests that the current evidence may not fully capture cross regional perspectives across WHO regions, particularly in the most resource constrained settings. Participant profiles demonstrate a dual emphasis: capacity building for healthcare providers and direct interventions for mothers. Most programs trained primary healthcare workers, nurses, midwives, and social workers, highlighting their central role in early identification and management of maternal mental health challenges. A smaller proportion focused on community cadres such as health volunteers and lay counselors, or directly engaged pregnant and postpartum women as participants in psychosocial education or support programs.

Methodologically, studies predominantly employed experimental or quasi-experimental designs aimed at evaluating training outcomes, complemented by formative and Delphi approaches for context specific curriculum development. The majority were implemented in primary or community healthcare settings, emphasizing local level integration. Training delivery was largely face-to-face, though hybrid and digital modalities are emerging as feasible alternatives to enhance accessibility. Across all interventions, course content primarily clustered around two domains: Clinical knowledge and screening skills for early detection of perinatal mental disorders, and Counseling and psychosocial support techniques that promote empathic and patient centered care. Evaluation strategies focused mainly on immediate learning outcomes such as knowledge, competence, and self-efficacy, with fewer studies extending assessment to behavioral, clinical, or system level impacts. Collectively, these patterns depict an expanding but regionally limited body of work that prioritizes practical, skills based MMH training within existing health service structures. This growing movement reflects increasing recognition of maternal mental health as a critical competency for non-specialist healthcare providers, though further efforts are needed to strengthen equity, scalability, and evaluation depth across diverse global contexts.
